# Bryophytes as Indicators of Disturbance in One of the Last Remnants of the Mountain Forests of El Oro Province, Ecuador

**DOI:** 10.3390/plants14020184

**Published:** 2025-01-11

**Authors:** Ángel Benítez, Richard Nagua, Jefferson Medina, Gregorio Lapo, Erika Yangua-Solano, Rolando Andrade-Hidalgo

**Affiliations:** 1 Biodiversidad de Ecosistemas Tropicales-BIETROP, Herbario HUTPL, Departamento de Ciencias Biológicas, Universidad Técnica Particular de Loja, San Cayetano s/n, Loja 1101608, Ecuador; naguarichard@gmail.com (R.N.); jeffersonmedinabenitez@gmail.com (J.M.); greglapo0525@yahoo.com (G.L.); enyangua@utpl.edu.ec (E.Y.-S.); 2 Departamento de Ciencias Jurídicas, Universidad Técnica Particular de Loja, Loja 1101608, Ecuador; rdandrade@utpl.edu.ec

**Keywords:** montane forests, non-vascular epiphytes, richness, composition, forest disturbance

## Abstract

Epiphytic bryophytes are an important component in terms of the diversity and functioning of montane forests known as biodiversity hotspots. Bryophytes are highly dependent on their external environments because they are sensitive to environmental changes related to disturbance, fragmentation, air pollution, and climate change. The richness and composition of bryophytes in remnants of primary and secondary forests were analyzed, where the richness and cover were recorded on trunk bases of 120 trees. Changes in species richness and diversity were analyzed using generalized linear models (GLMs), and changes in species composition, using multivariate analysis. A total of 57 bryophyte species (36 liverworts and 21 mosses) were recorded in trunk bases. For the first time, 19 new liverworts for the province of El Oro are reported. The richness and diversity of bryophyte species decrease in disturbed forests when compared to primary forests, with a marked decrease in species less adapted to conditions of high light (shade epiphytes). In the same line, species composition is different in each type of forest, where bryophytes with high humidity requirements were abundant in primary forests. This study confirms that forest disturbance is a key factor in determining not only the number of species but also the composition of bryophyte species. The maximum tree diameter and primary forest remnants are important factors in the conservation of sensitive bryophyte species at the base of trees in one of the last remnants of mountain forests in El Oro Province, Ecuador.

## 1. Introduction

Land-use change is a major contributor to global biodiversity loss [1,2], including the megadiverse tropical montane forest [3,4]. These forest changes are associated with habitat loss, fragmentation, and forest disturbance [5,6,7], and thus, previous studies have shown the negative effects of disturbance in these forests on the diversity of several plant and animal species [8,9,10,11].

Montane forests are ecosystems of great importance because they harbor high diversity; therefore, they are considered a global conservation priority, being recognized as one of the global biodiversity hotspots [12,13]. Due to the presence of climatic factors and geological and edaphic conditions [14], these ecosystems intervene in water regulation, climate sustainability and constitute an important carbon sink [14,15]. Despite this, these forests are currently reduced to small fragments due to deforestation, mainly for agricultural and livestock activities [16,17].

Epiphytic organisms in montane forests are well represented [18,19,20], where a characteristic group comprises the bryophytes that constitute an important component within these ecosystems [21,22,23,24,25]. In addition, due to their different forms of life, diversity, and biomass, they intervene in water regulation, stabilize the soil, and retain moisture [19,26]. Because they are organisms that lack water regulation mechanisms (pohiquilohydric), they are used as indicator species of environmental changes such as the alteration of ecosystems [25,27,28], air pollution [29,30], and climate change [31,32]. Epiphytic bryophytes are limited by forest structure (tree cover, age, size of the host tree, bark texture, and pH of the bark) and microclimatic factors [24,25,26,33]; therefore, previous studies have shown that forest disturbance reduces diversity in primary forests when compared to secondary forests and plantations [19,24,25,28,34].

In southern Ecuador, montane forests are exposed to high anthropogenic pressures related to agricultural activities, forestry, and mining practices [17,35]. In addition, studies on the diversity of bryophytes associated with forest disturbance in the Southern Andes of Ecuador are very limited and restricted to the province of Loja [24,25,28]. The Casacay river sub-basin provides water resources for extensive agricultural areas, including banana plantations, and serves major cities in El Oro Province, where the montane forests, specifically of the Casacay river sub-basin, are subjected to deforestation, agricultural activities, forest fires, and the introduction of exotic plants, for example, *Pinus patula* Schiede ex Schltdl. & Cham. plantations. As result, the Casacay sub-basin has shown a significant decrease in natural forest areas, such as montane forests, from 56% in 1990 to 29.12% in 2022, while agricultural land has increased from 35.27% in 1990 to 59.24% in 2022 [36,37]. However, studies using epiphytes (e.g., lichens) as indicators of disturbance in the province of El Oro have only been carried out in the dry forests of the Reserva Ecológica Arenillas [38]. Therefore, this is the first study to use epiphytic bryophytes as indicators of disturbance in montane forests in the province of El Oro.

We aimed to quantify how anthropogenic disturbance processes affect bryophyte diversity in a tropical montane forest of southern Ecuador, an ecosystem recognized as a biodiversity hotspot [12]. While most of our knowledge on this topic comes from Loja Province, empirical evidence on the bryophyte response to forest disturbance remains limited for the montane forests of El Oro Province montane forests. To address this, we asked the following questions: (a) Do forests with different levels of disturbance affect bryophyte diversity in a montane rainforest? (b) Do bryophytes show similar species composition in responses to forest disturbances?

## 2. Results and Discussion

**Figure 1:**
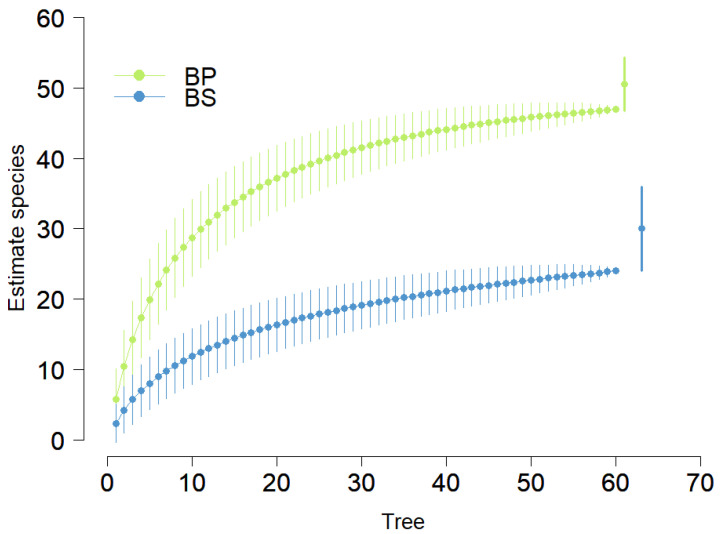
Accumulation curve for the two types of forest in El Oro Province, the southern region of Ecuador. BP = primary forest, BS = secondary forest.

We registered 57 species distributed in 36 liverworts and 21 mosses in 120 trees (Table A1). Moreover, we report 19 new liverwort species for the province of El Oro (Table A1), where 82 species were known to date; therefore, the number of liverwort species reached a total of 101 species. The 57 species of bryophytes reported in this study are considered similar when compared to the study carried out by Medina et al. [25], which recorded 55 species in secondary montane forests, the 67 species of bryophytes reported by Benítez et al. [24], and finally the 86 species of bryophytes (49 liverworts and 37 mosses) reported from a montane forest in Panamá [39], which indicated a high diversity of epiphytic bryophytes in tree trunks of the montane forests that are considered biodiversity hotspots [12]. This information is corroborated by the accumulation curve and the Chao 2 estimator, which indicated a higher number of estimated species for the primary forest compared to the secondary forest (Figure 1). In agreement with our results, Fernández-Prado [7] found the highest number of epiphytic cryptogams in montane forests compared to disturbed forests, for example, monospecific secondary forests of *Alnus acuminata* Kunth.

**Figure 2:**
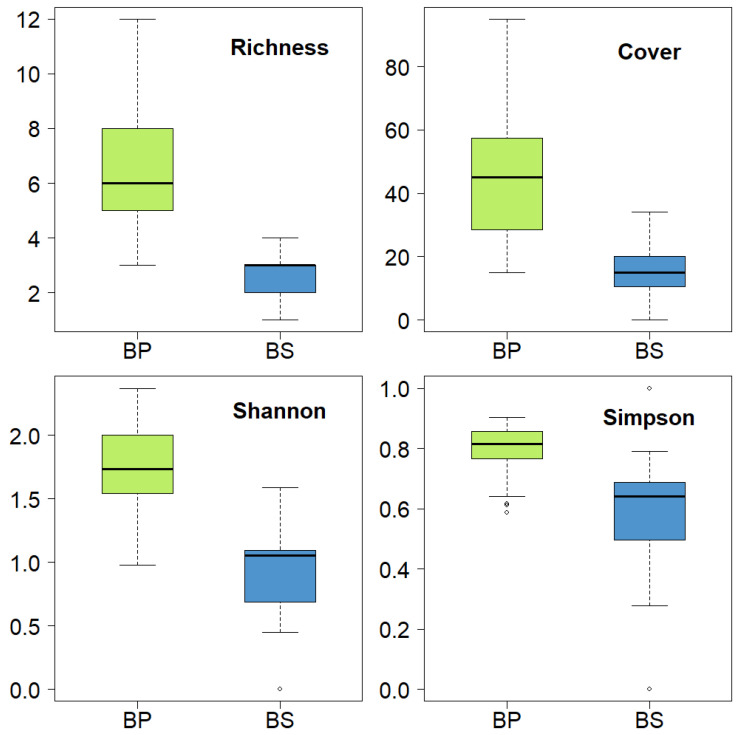
Box plot on richness, abundance, and diversity indices (Shannon and Simpson) for the two types of forests (BP, BS) in El Oro Province, the southern region of Ecuador.

The box plots indicated that richness, cover, and the Simpson and Shannon–Weaver indices were higher in primary forests when compared to secondary forests (Figure 2).

The GLM showed that the primary forest and DBH positively influenced the richness, cover, and diversity of species; conversely, the secondary forest negatively influenced the richness, cover, and diversity of species (Table 1). Our results show significant changes in richness, diversity, and composition between the two forest types; these changes are related to disturbance in the secondary forest, which implies microclimatic changes. In this context, previous studies have reported the negative effects of forest disturbance in bryophytes diversity in tropical montane forests [6,24,25,28,39,40]. For example, Gradstein and Sporn [6] showed changes and losses in the richness and diversity of the bryophytes of tropical rainforests due to human activities in Bolivia, Ecuador, Costa Rica, and Indonesia.

The richness and diversity of bryophytes were higher in primary forests than in secondary forests, where there is less moisture available. A similar pattern was found by Acebey et al. [34], who found that bryophyte diversity was affected by forest disturbance. Likewise, concerning the montane forests of Ecuador, Nöske et al. [28] and Benítez et al. [24] pointed out that the disturbance of the forests significantly affects the richness and diversity of bryophytes caused by the decrease in tree cover, smaller size of the host trees, and greater incidence of light.

**Figure 3:**
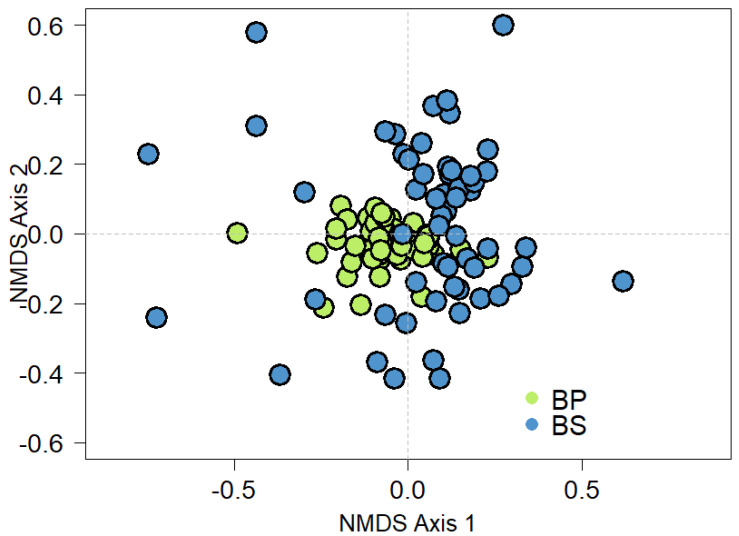
Non-parametric multidimensional scaling (NMDS) analysis of species composition in the two forest types in El Oro Province, the southern region of Ecuador. Primary forests (green circles) and secondary forests (blue circles).

NMDS analysis indicated a clear separation between the bryophyte communities between primary and secondary forests (Figure 3).

PERMANOVA indicated that the forest type and DBH significantly influenced the composition of bryophyte communities, where forest type explained 7%, followed by DBH with 1% (Table 2). For species composition, a significant change was obtained between primary and secondary forests; this pattern has been found in several studies [28,34,38]. For example, primary forests present favorable climatic conditions for the survival of genera of bryophytes such as *Herbertus*, *Plagiochila, Syzygiella*, and *Leiomela* that need very high hydration levels [24,25,34]. Similar studies have indicated that these shade species are present in humid and mature forests, with dense tree cover and high humidity that favor high levels of hydration; therefore, the low presence or absence of these species would be related to the disturbance or fragmentation of forests [19,21,26]. On the contrary, secondary forests with less tree cover are dominated by species of the genera *Frullania, Radula*, and *Marcomitriun* that have adapted to conditions of greater incidence of light [24,25,34].

Finally, tree diameter influenced the richness, diversity, and composition of bryophytes, which is in agreement with some studies carried out in montane forests, where a greater diameter of trees is related with greater surface area for bryophyte colonization processes [24,40].

## 3. Materials and Methods

### 3.1. Study Area

**Figure 4:**
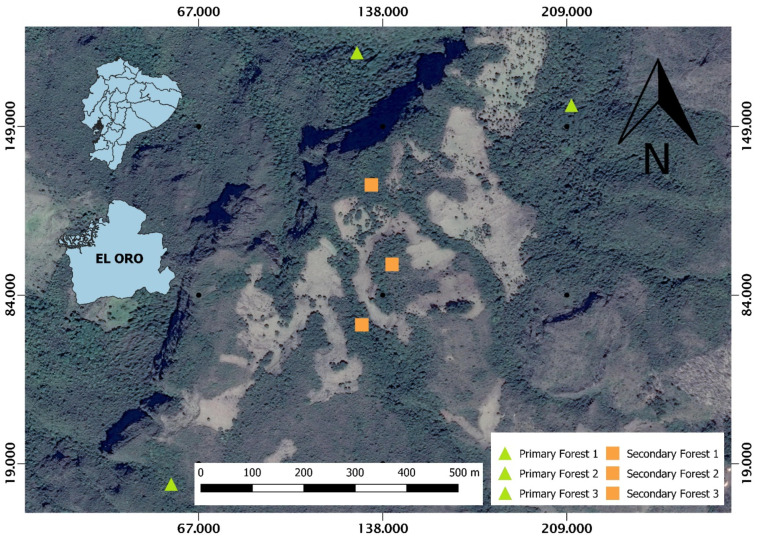
Study area shows the Casacay river sub-basin in El Oro Province, the southern region of Ecuador. BP = primary forest, BS = secondary forest.

This study was carried out in the upper part of the Casacay river sub-basin of the Chilla canton, province of El Oro, at an altitude that oscillates between 2705 and 2865 masl at the coordinates 3.4784° S and 79.6192° W, with an average annual temperature of 9 to 16 °C and average annual rainfall of 1160 mm (“Plan de manejo de la Subcuenca del río Casacay”) 2011 [36,37]. In the area, we selected remnants of primary and secondary forests (Figure 4).

**Figure 5:**
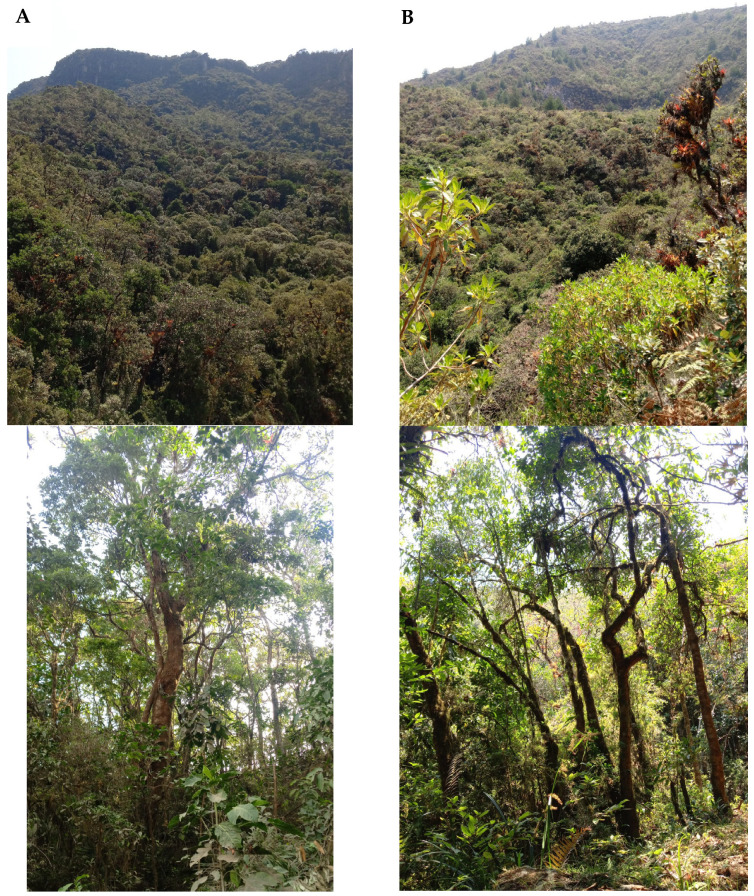
Two types of forest (primary forest and secondary forest) in the Casacay river sub-basin in El Oro Province, the southern region of Ecuador. **A** = primary Forests, **B** = secondary forests.

Primary Forests (Figure 5A): These are ecosystems that have large trees, shrubby vegetation, and stable ecological processes and lack human activities, [24,41,42]. Secondary forests (Figure 5B): They originate as a result of human activities, in our case, specifically due to forest fires and selective logging. They are characterized by a decrease in tree cover, high incidence of light, and low humidity [28,43].

### 3.2. Design and Data Collection

The present study was carried out in two types of forest (primary forest and secondary forest). Three fragments were selected for each type of forest; in each fragment, 5 plots of 5 × 5 m were made, and 4 random trees were selected for each plot, in which the DBH (diameter at breast height) greater than 10 cm was taken (120 trees). Epiphytic bryophytes were sampled using a 20 × 30 cm grid [24], at a height of 1 m in trunk bases. To minimize the edge effect, the samples trees were taken from 100 m from the beginning of the forest [44]. The samples were collected in paper bags (a bag per plot) to later be identified using general and specialized bryophytes keys [45,46,47,48] and deposited in the HUTPL herbarium (collection of bryophytes and lichens).

### 3.3. Data Analysis

To determine the diversity of species, samples were analyzed using the specific richness, coverage, and the Simpson and Shannon–Weaver indices. Likewise, the effects of the forest type and DBH on species richness at the tree level were analyzed using a generalized linear model (GLM).

To analyze the changes in the composition of the epiphyte bryophyte communities, a non-metric multidimensional scaling (NMDS) analysis was carried out with species cover values at the tree level in the two forest types. In addition, to determine the effects of forest type and DBH on the composition of the communities, a multivariate analysis based on permutations (PERMANOVA) was used. These analyzes were performed with the statistical program R v.1.1.456 [49] with the “vegan” package [50].

## 4. Conclusions

The diversity of epiphytic bryophytes was negatively affected by forest disturbance, with primary forests showing greater species richness and bryophyte communities differing between the two forest types. We also report 19 new liverwort species for the province of El Oro, bringing the total number of species to 101. This study confirms that forest disturbance is a key factor in determining not only bryophyte diversity but also bryophyte communities, with large trees and primary forest remnants being important factors in the conservation of bryophyte species in one of the last remnants of mountain forests in El Oro Province, Ecuador. Epiphytic bryophytes are effective indicators of disturbance in the montane forests of the Casacay river sub-basin, which will allow for establishing management and conservation strategies for these forests. We recommend that future research prioritize examining the relationship between microclimatic factors (e.g., light, moisture and bark characteristics) and the taxonomic and functional diversity of bryophytes in response to climate change, disturbance, and fragmentation in the montane forests of the Casacay river sub-basin.

## Figures and Tables

**Table 1:** Results of the generalized linear model (GLM) on richness, coverage, and Shannon and Simpson indices based on BP and BS in El Oro Province, the southern region of Ecuador.

**Richness**	**Estimator**	**St**	**t**	***p*-Value**
BP	0.898538	0.230456	3.899	<0.0001
BS	−0.554814	0.119361	−4.648	<0.0001
DBH	0.037073	0.008361	4.434	<0.0001
**Coverage**				
BP	88.641	68.336	1.297	0.197
BS	−178.702	32.929	−5.427	<0.0001
DBH	13.939	0.2564	5.437	<0.0001
**Shannon**				
BP	0.754154	0.171721	4.392	<0.0001
BS	−0.521098	0.082747	−6.298	<0.0001
DBH	0.037801	0.006443	5.867	<0.0001
**Simpson**				
BP	0.612129	0.082573	7.413	<0.0001
BS	−0.170701	0.039789	−4.290	<0.0001
DBH	0.007163	0.003098	2.312	0.0225

**Table 2:** Results of PERMANOVA on three factors in the composition of species at the plot, fragment, and forest level in El Oro Province, the southern region of Ecuador.

Factor	Df	SS	R^2^	F	*p*-Value
Forest	1	2101.0	0.07991	103.288	0.001
DBH	1	390.6	0.01486	19.204	0.005
Residuals	117	23799.2	0.90523		
Total	119	26290.9	100.000		

Df = degrees of freedom; SS = sum of squares; R^2^ = coefficient of variation; F = F statistics.

## Data Availability

The original contributions presented in the study are included in the article; further inquiries can be directed to the corresponding author.

## Appendix A

**Table A1:** Species of bryophytes from the Casacay river sub-basin of the Chilla canton in El Oro Province, the southern region of Ecuador.

Species	BP	BS	New Report for El Oro
**Liverworts**			
*Bazzania longistipula* (Lindenb.) Trevis	x		x
*Caudalejeunea lehmanniana* (Gottsche et al.) A.Evans	x		x
*Frullania peruviana* Gottsche	x	x	
*Frullania dusenii* Steph		x	x
*Herbertus acanthelius* Spruce	x		x
*Herbertus pensilis* Spruce	x		x
*Herbertus sendtneri* (Nees) Lindb.	x		x
*Lejeunea cerina* (Lehm. & Lindenb.) Gottsche et al.	x	x	
*Lejeunea* aff. *laetevirens Nees & Mont*.	x		
*Lejeunea* sp. 1	x	x	
*Lejeunea* sp. 2	x		
*Leiomitra sprucei* (Steph.) T.Katagiri	x		x
*Lepicolea pruinosa* (Taylor) Spruce	x		x
*Lepidozia cupressina* (Sw.) Lindenb.	x		x
*Leptoscyphus intermedius* Grolle	x	x	x
*Lepidolejeunea* sp.	x	x	
*Marchesinia brachiata* (Sw.) Schiffn.		x	x
*Metzgeria crassipilis* (Lindb.) A.Evans	x	x	
*Metzgeria myriopoda* Lindb.		x	x
*Microlejeunea bullata* (Taylor) Steph.	x		x
*Omphalanthus filiformis* (Sw.) Nees	x		
*Omphalanthus ovalis* (Lindenb. & Gottsche) Gradst.		x	
*Plagiochila aerea* Taylor	x		
*Plagiochila bifaria* (Sw.) Lindenb.	x	x	x
*Plagiochila* aff. *rutilans* Lindenb	x		
*Plagiochila raddiana* Lindenb	x	x	
*Plagiochila* sp. 1		x	
*Plagiochila* sp. 2	x		
*Porella squamulifera* (Taylor) Trevis.	x	x	x
*Porella swartziana* (F.Weber) Trevis.	x		
*Prionolejeunea decora* (Taylor) Steph.	x		
*Radula pallens* (Sw.) Nees ex Mont.	x		
*Radula voluta* Taylor		x	x
*Scapania portoricensis* Hampe & Gottsche	x	x	x
*Syzygiella manca* (Mont.) Steph.	x		x
*Syzygiella perfoliata* (Sw.) Spruce	x		x
**Mosses**			
*Hemiragis aurea* (Brid.) Kindb.	x		
*Helicodontium capillare* (Hedw.) A.Jaeger	x		
*Leiomela bartramioides* (Hook.) Paris	x		
*Leptodontium pungens* (Mitt.) Kindb.		x	
*Macromitrium punctatum* (Hook. & Grev.) Brid.	x	x	
*Macromitrium richardii* Schwägr.	x	x	
*Macromitium* sp.	x	x	
*Meteoridium tenuissimum* (Hook. & Wilson) M.A.Lewis	x	x	
*Meteoridium remotifolium* (Müll. Hal.) Manuel	x		
*Octoblepharum pulvinatum* (Dozy & Molk.) Mitt.	x		
*Neckeropsis undulata* (Hedw.) Reichardt	x	x	
*Pleurozium schreberi* (Brid.) Mitt.	x		
*Orthotrichum steerei* Lewinsky	x		
*Porotrichum lancifrons* (Hampe) Mitt.	x		
*Prionodon densus* (Hedw.) Müll. Hal.	x	x	
*Rhodobryum beyrichianum* (Hornsch.) Müll. Hal.	x		
*Sematophyllum erythropodium* Mitt.	x		
*Sematophyllum* sp.	x	x	
*Squamidium nigricans* (Hook.) Broth.	x	x	
*Syrrhopodon* sp.	x		
*Thuidium peruvianum* Mitt.	x		
